# Differences in Immune Characteristics and Related Gene Expression in Spleen among Ningxiang, Berkshire Breeds and Their Hybrid Pigs

**DOI:** 10.3390/genes15020205

**Published:** 2024-02-04

**Authors:** Gang Song, Yuebo Zhang, Hu Gao, Yawei Fu, Yue Chen, Yulong Yin, Kang Xu

**Affiliations:** 1 College of Animal Science and Technology, Hunan Agricultural University, Changsha 410128, China; songgang19971109@163.com (G.S.); ybzhangfd@hunau.edu.cn (Y.Z.); gaohu_20190008@163.com (H.G.); fuyw2020@163.com (Y.F.); 2 Guangdong Laboratory for Lingnan Modern Agriculture, Guangzhou 510642, China; 18837025618@126.com; 3 Key Laboratory of Agroecological Processes in Subtropical Region, Institute of Subtropical Agriculture, Chinese Academy of Sciences, Changsha 410125, China; 4 Hunan Provincial Key Laboratory of the Traditional Chinese Medicine Agricultural Biogenomics, Changsha Medical University, Changsha 410219, China

**Keywords:** spleen, immune difference, RNA-Seq, mRNA, pig breed, hybrid pig

## Abstract

To investigate the differential immunology in Ningxiang and Berkshire pigs and their F_1_ offspring (F_1_ offspring), physiological and biochemical indicators in the plasma and spleen were analyzed. Then, transcriptomic analysis of the spleen identified 1348, 408, and 207 differentially expressed genes (DEGs) in comparisons of Ningxiang vs. Berkshire, Berkshire vs. F_1_ offspring, and Ningxiang vs. F_1_ offspring, respectively. In Ningxiang vs. Berkshire pigs, the gene ontology (GO) and the Kyoto Encyclopedia of Genes and Genomes (KEGG) analysis indicated that the DEGs included *CD163*, *MARCO*, *CXCL14*, *CCL19*, and *PPBP*, which are associated with immunity. GO and KEGG analyses were also conducted comparing F_1_ offspring and their parents. The DEGs, including *BPIFB1*, *HAVCR2*, *CD163*, *DDX3X*, *CCR5*, and *ITGB3*, were enriched in immune-related pathways. Protein–protein interaction (PPI) analysis indicated that the *EGFR* and *ITGA2* genes were key hub genes. In conclusion, this study identifies significant immune DEGs in different pig breeds, providing data to support the exploration of breeding strategies for disease resistance in local and crossbred pig populations.

## 1. Introduction

Pigs (*Sus scrofa*) play a significant role in both agriculture and medicine. In contrast to the European native Berkshire pig or hybrid pig breeds, indigenous Chinese breeds have the advantages of adaptability to environmental conditions, climate adaptation, and enhanced disease resistance, alongside commendable meat quality [1]. The indigenous Chinese Ningxiang pig has the characteristics of strong adaptability, high disease resistance, and tolerance for coarse feed. Although indigenous Chinese breeds are thought to have developed high disease resistance through evolution, experimental evidence for this has been lacking [2]. Therefore, the immune characteristics of Ningxiang pigs and European native Berkshire and hybrid pigs remain to be determined. The spleen is a crucial immune organ in animals and is the largest component of the lymphatic system, which has immune response functions [3]. The spleen serves as a hub for both the innate and adaptive immune processes. Crucially, a multitude of mononuclear phagocytes can be found in the spleen. These cellular entities possess the ability to detect pathogens and cellular stress, eradicate deceased cells and foreign intruders, uphold the tissue balance, regulate inflammatory reactions, and play significant roles in adaptive immunity [4].

Transcriptome sequencing has recently become an increasingly popular method for investigating transcriptomes in various areas, including biological, medical, clinical, and pharmaceutical research. In the present study, the immune performance of indigenous Chinese breeds, the European native Berkshire pig, and their hybrid breeds were examined by assessing and contrasting plasma physiological and biochemical markers in Ningxiang pigs, Berkshires, and their F_1_ offspring (F_1_ offspring). Additionally, we conducted transcriptome sequencing on spleen tissues, aiming to pinpoint immune-related differentially expressed genes (DEGs) and understand the immune differences between the breeds.

## 2. Materials and Methods

### 2.1. Animals and Sample Collection

This study included 14 Berkshires, 13 Ningxiang pigs and 12 F_1_ offspring (all from the Ningxiang Pig Farm, Hunan Dalong Animal Husbandry Technology Co., LTD., Changsha, China). These pigs were all female fattening pigs. In addition, there were no epidemiological outbreaks on the pig farm. During the growth and fattening stages, each pen housed a group of 10–12 pigs. The pigs were kept in standard environmental conditions without controlled temperature or lighting. They ate a standard daily diet and had free access to water. Adhering to the established protocol, healthy pigs of similar weight were randomly selected from each breed for subsequent analysis. Before slaughtering, the pigs underwent fasting. The mean body weights at slaughter were 98.1 kg for Ningxiang pigs, 118.61 kg for Berkshire pigs and 115.8 kg for F_1_ offspring, slaughtered on the same day. After slaughtering, all spleen samples used for this test were obtained from a transverse section which was made at the largest extension of the spleen, showing red and white pulp. This method allows for obtaining white pulp for study purposes [5]. Spleen samples were stored in liquid nitrogen immediately after collection and kept in a −80 °C refrigerator for one week until analysis.

### 2.2. Measurement of Physiological and Biochemical Indicators

#### 2.2.1. Determination of Immunological Indicators in Plasma and Spleen by ELISA

Plasma and spleen samples were collected and subjected to immunological assessments using the ELISA method. Antibody detection was conducted in line with reagent kit instructions, with subsequent calculation of the actual concentration of the sample. Immune parameters within the plasma, such as IgA (MS4658-A), IL-2 (MS4573-A), IL-6 (MS4570-A), and IFN-γ (MS4564-A), were quantified. Moreover, immune markers within the spleen, including IL-4 (MS4571-A), IL-6 (MS4570-A), IL-17 (MS4529-A), IFN-γ (MS4564-A), and TNF-α (MS4535-A), were measured. When preparing spleen tissue for ELISA testing, the sample was placed in a clean mortar and grind with liquid nitrogen. A spleen sample of 0.1g was placed into a centrifuge tube, PBS was added to mix it well, and the supernatant was collected. Samples diluted 30 times with the stock solution were selected by the BCA protein concentration determination kit through pre-experiment for concentration normalization. Samples with normalized concentrations were used for ELISA. All ELISA kits were procured from Shanghai Lange Biotechnology Co. (Shanghai, China) Each indicator has a different detection range and sensitivity. The detection range and sensitivity of ELISA kits are shown in Table 1.

#### 2.2.2. Measurement of Plasma Biochemical Indicators

A fully automated Roche cobas c311 biochemical analyzer was used to measure 13 standard indicators, including albumin (ALB), alanine aminotransaminase (ALT), and alkaline phosphatase (ALP), across a total of 42 pigs.

### 2.3. Preparation of Samples, Construction of RNA Libraries, and Sequencing

The blood collection procedure used a needle for blood collection into anticoagulant tubes containing either ethylenediaminetetraacetic acid (EDTA) or heparin. After blood collection, the plasma was separated and frozen until further analysis. RNA was extracted from spleen tissues by employing Trizol reagent (Invitrogen, Waltham, MA, USA). After quality assessment, RNA samples were sequenced by constructing libraries on the Illumina NovaSeq 6000 platform (Illumina, San Diego, CA, USA). Quality control was performed on the raw Illumina sequences using FASTQC software (version 0.11.7) to obtain clean reads for subsequent analysis. The paired-end reads obtained by Illumina sequencing were compared with the reference genome (Sus scrofa 11.1) using Hisat2 software (version 2.0.5).

### 2.4. Differential Expression Analysis and Functional Enrichment

Differential expression analysis in spleen tissue was conducted using DESeq2. The criteria for differential gene screening were log_2_(Fold Change) > 1 and false discovery rate (FDR) < 0.05. Enrichment analysis of the DEGs was conducted using the clusterProfiler R package. The *p*-values were subjected to adjustment by the FDR method.

### 2.5. Quantitative Real-Time PCR (qRT-PCR)

Tissue RNA was extracted by the trizol (Invitrogen) conventional method, and cDNA was obtained by reverse transcription of RNA with the *Evo M-MLV* RT Mix Kit with gDNA Clean for qPCR Ver. 2 (AG Scientific, San Diego, CA, USA). Primers were designed for the target genes using NCBI and synthesized by Tsingke Biotechnology Co., Ltd. (Beijing, China). RT-qPCR was carried out using a qPCR kit (AG Scientific, San Diego, CA, USA). We used the β-actin reference gene because β-actin is stably expressed in different tissues with good uniformity (Table 2). The relative mRNA expression was analyzed using the 2^−ΔΔCT^ method.

### 2.6. Statistical Analysis

Statistical analyses were performed and visualized using SPSS 26.0 and GraphPad Prism 8.0 software. The results are expressed as mean ± standard deviation. The statistical test was a *t*-test with a statistical significance level of 0.05. Enrichment analysis was carried out using R packages (version 4.3.0).

## 3. Results

### 3.1. Plasma and Spleen Physiological and Biochemical Indices

We analyzed the physiological and biochemical indicators related to immunity in Ningxiang pigs, Berkshires, and F_1_ offspring (Table 3). The IgA (immunoglobulin A) and IFN-γ (interferon-γ) levels in the plasma from Ningxiang pigs and F_1_ offspring were significantly higher than those of Berkshires (*p* < 0.05). The IL-2 (interleukin-2) levels in the plasma of Ningxiang pigs and Berkshires were significantly higher than that of F_1_ offspring (*p* < 0.05). The IL-6 (interleukin-6) level in the plasma of Ningxiang pigs was significantly higher than those of Berkshires and F_1_ offspring (*p* < 0.05).

In the spleen, the levels of IL-4 (interleukin-4) and IL-17 (interleukin-17) in Ningxiang pigs were significantly higher than those in Berkshires and F_1_ offspring (*p* < 0.05). The IL-6 and IFN-γ levels in the spleen of Ningxiang pigs were significantly higher than that of Berkshires, and the IFN-γ level in the spleen of F_1_ offspring was significantly higher than that of Berkshires (Table 3).

The ALT, glucose (GLU), cholesterol (CHOL), and low-density lipoprotein (LDL-C3) levels in the plasma of Ningxiang pigs were significantly higher than those of Berkshire pigs. Conversely, the ALB levels in the plasma of Ningxiang pigs were significantly lower than those of Berkshire pigs. Compared with the F_1_ offspring, the alkaline phosphatase (ALP) levels in the plasma of both parental pig breeds were significantly higher (Table 4).

### 3.2. Annotation of DEGs and Their Functions in Different Pig Breeds

**Figure 1:**
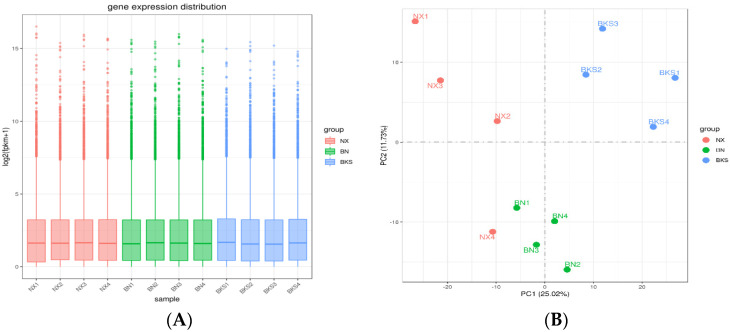

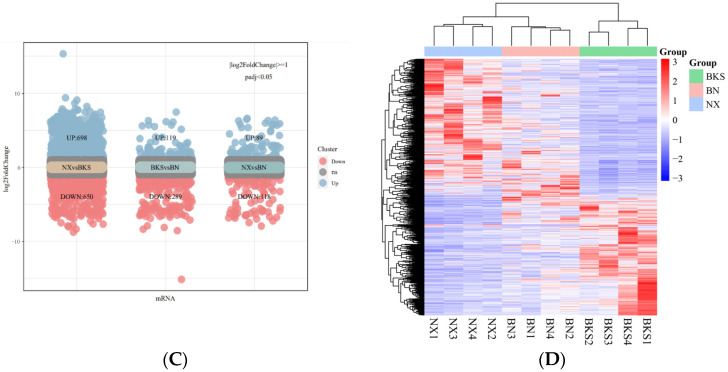
(**A**) The gene expression distribution of all samples. (**B**) Principal component analysis (PCA) diagram of all samples. (**C**) Differential genes in the three groups. (**D**) Heatmap showing the clustering of DEGs, with sample names on the horizontal axis and normalized differential gene FPKM values on the vertical axis.

According to the data, there was a total of 14,586 genes in all pig groups. At the same time, we analyzed that the common genes were 12,015. To explore DEGs between groups, we conducted differential gene expression analysis. Figure 1A shows the distribution of gene expression levels across the samples depicted by a boxplot. Comparing Ningxiang and Berkshire pigs, 1348 DEGs were identified, of which 698 were upregulated and 650 were downregulated. The comparison between Ningxiang pigs and F_1_ offspring revealed 207 DEGs. Of these, 89 genes were upregulated and 118 genes were downregulated. Additionally, when comparing Berkshire pigs and F_1_ offspring, there were 417 DEGs, including 119 upregulated genes and 298 downregulated genes (Figure 1C). Figure 1D shows a clustering heatmap that displays the expression patterns between groups.

### 3.3. GO Enrichment Analysis

**Figure 2:**
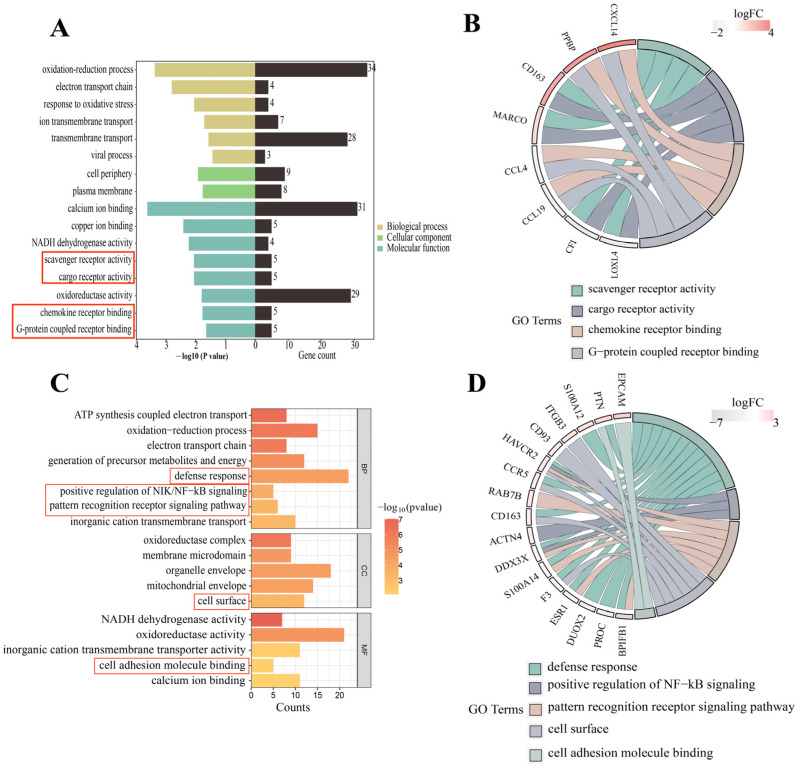
(**A**) GO analysis of the DEGs in the comparison of Ningxiang pigs vs. Berkshires. (**B**) GO functional classification of DEGs in the comparison of Ningxiang pigs vs. Berkshires. (**C**) GO analysis of DEGs in F_1_ offspring of Ningxiang and Berkshires pigs (F_1_ offspring) and parental pigs. (**D**) GO functional classification of DEGs in F_1_ offspring and parents. The red boxes in (**A**,**C**) are labelled with immune-related terms.

Gene ontology (GO) term analysis showed that there were 36 statistically significant terms for Ningxiang pigs compared with Berkshires. The significantly upregulated terms were mainly involved in G-protein coupled receptor binding, scavenger receptor activity, cargo receptor activity, ion transmembrane transport, and chemokine receptor binding. The significantly downregulated terms in Ningxiang pigs were mainly related to the regulation of multicellular organismal processes and response to oxidative stress (Figure 2A). Notably, the GO annotations of G protein-coupled receptor binding, cargo receptor activity, chemokine receptor binding, and scavenger receptor activity are primarily connected to immune responses and immune reactions. Gene annotation revealed that genes such as *CD163* (cluster of differentiation 163) were related to these two molecular function (MF) terms (Figure 2B).

In the genetic test, we firstly analyzed Ningxiang vs. Berkshire pigs, F_1_ offspring vs. Ningxiang pigs and F_1_ offspring vs. Berkshire pigs. Then, we compared F_1_ offspring to Ningxiang and Berkshire pigs mainly to comprehensively study and analyze the differences in genetic traits between crossbred offspring and both parents. In comparison to the parental pigs, GO term analysis showed 56 statistically significant terms in the F_1_ offspring. Specifically, within the biological process (BP) category, the annotation results were primarily associated with ATP metabolic processes, oxidative phosphorylation, the electron transport chain, defense response, cellular respiration, positive regulation of NF-κB signaling, and the pattern recognition receptor signaling pathway. In the cellular component (CC) category, annotations related to the cell surface and mitochondrial respiratory chain were particularly prevalent. In the MF category, the annotations encompassed activities such as NADH dehydrogenase activity, oxidoreductase activity, and cell adhesion molecule binding (Figure 2C). Of note, GO terms related to the immune response included the defense response, positive regulation of NIK/NF-κB signaling, and cell surface and cell adhesion molecule binding functions (Figure 2C). On gene annotation, we found that the genes *F3* (coagulation factor III)*, CD163*, *DUOX2* (dual oxidase 2), *PTN* (pleiotrophin), *CCR5* (C-C chemokine receptor type 5), *PROC* (protein C), *S100A12* (S100 calcium-binding protein A12), and *ACTN4* (actinin α4) were annotated to the immune response-related BP terms. *EPCAM* (epithelial cell adhesion molecule), *HAVCR2* (hepatitis A virus cellular receptor 2), *F3*, *ITGB3* (integrin subunit β3), *CD163*, *CD93* (complement component 3d receptor 1), and *CCR5* were involved in cell surface. The genes *EPCAM*, *ITGB3*, *PTN*, *S100A14* (S100 calcium-binding protein A14), *CD93*, *DUOX2*, *ACTN4*, *PROC*, and *S100A12* were involved in the two immune-related MF terms. The genes *DDX3X* and *HAVCR2* were commonly enriched in these three immune-related BP processes, and the genes *BPIFB1* (BPI fold-containing family B, member 1), *S100A14*, *CH3L1* (chitinase 3 like 1), *RAB7B* (RAS oncogene family member), and *ESR1* (estrogen receptor 1) were commonly linked to two of the immune-related BP processes (Figure 2D).

**Figure 3:**
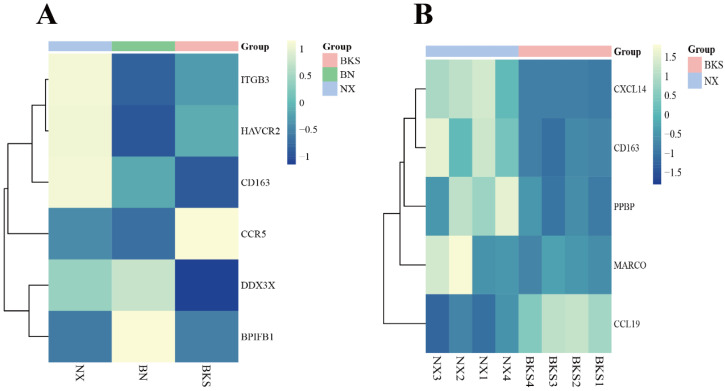
(**A**) Heatmap of clustering expression of screened DEGs in Ningxiang pigs, Berkshires, and F_1_ offspring. (**B**) Heatmap of clustering expression of screened DEGs in Ningxiang and Berkshire pigs.

Regarding the immune terms and pathways in this study, we screened for several immune DEGs. In comparison to Berkshires, the *CXCL14*, *CD163*, *PPBP*, and *MARCO* genes were upregulated in Ningxiang pigs, and *CCL19* was downregulated in Ningxiang pigs (Figure 3A). Compared with the parents, the *DDX3X* and *BPIFB1* genes were upregulated in F_1_ offspring, and *ITGB3, HAVCR2*, and *CCR5* were downregulated in F_1_ offspring. The expression level of *CD163* was intermediate between Ningxiang and Berkshire pigs (Figure 3B).

### 3.4. KEGG Enrichment Analysis

**Figure 4:**
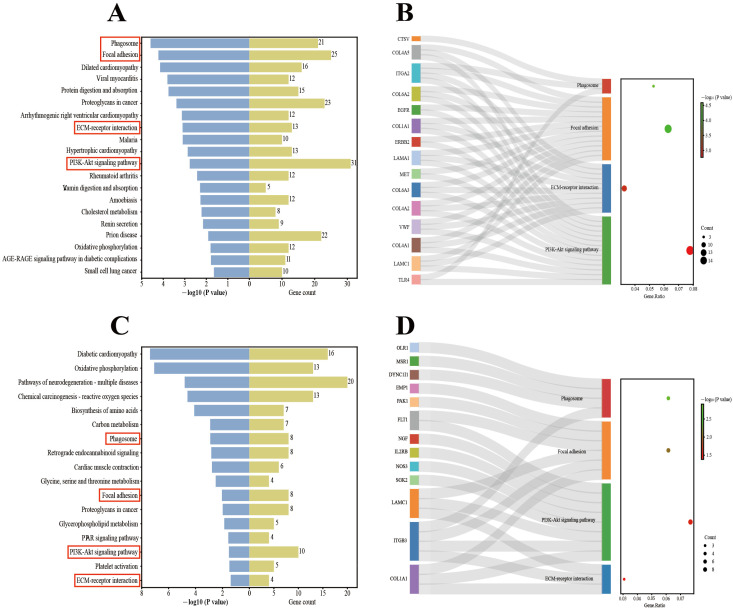
(**A**,**B**) KEGG enrichment analysis of DEGs in the comparison of Ningxiang pigs vs. Berkshires. (**C**,**D**) KEGG enrichment analysis of DEGs in F_1_ offspring and parents. The red boxes in (**A**,**C**) are labelled with immune-related pathways.

For Berkshires vs. Ningxiang pigs, our analysis focused on the top 20 KEGG pathways. The results showed that the enriched pathways included the immune-related pathways (Figure 4A). In addition, 15 genes, including *ITGA2*, *TLR4*, and *EGFR*, were enriched in at least two of the above pathways (Figure 4B).

For the F_1_ offspring vs. parents, our analysis also focused on the top 20 pathways. Of the results, the enriched pathways included immune-related pathway (Figure 4C). Furthermore, there were 13 genes that were enriched in at least two of the above pathways (Figure 4D).

### 3.5. PPI Network and Hub Gene Identification

**Figure 5:**
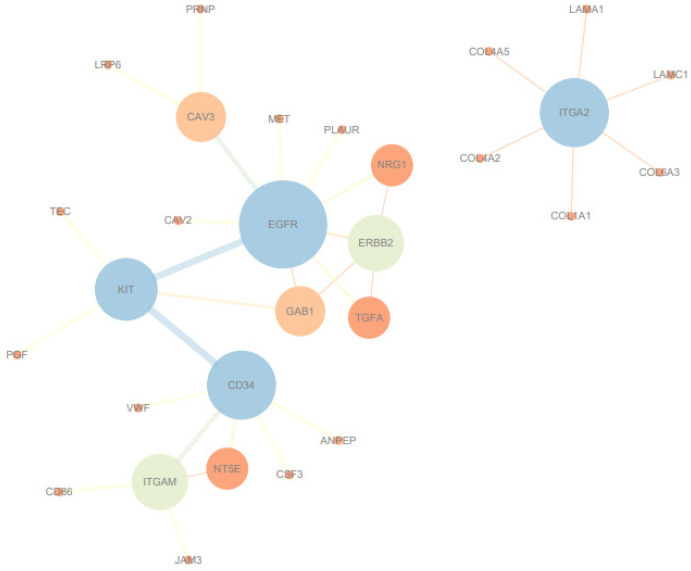
PPI network and identification of hub genes of the DEGs in Ningxiang pigs vs. Berkshires.

**Figure 6:**
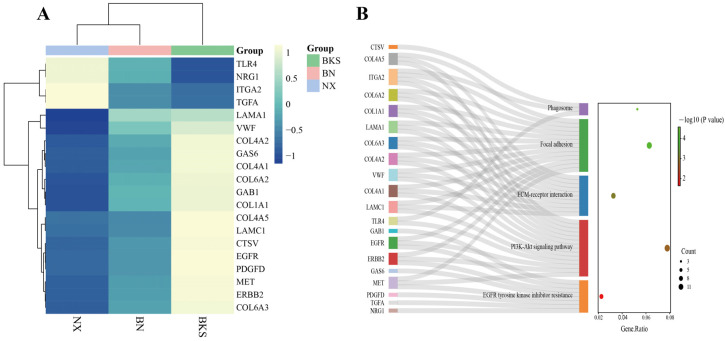
(**A**,**B**) The clustering heat map of key gene expression and the pathways involved in key genes.

When comparing Ningxiang pigs with Berkshires, the PPI network analysis identified *EGFR* and *ITGA2* as hub genes (Figure 5). *EGFR* is involved in the PI3K–Akt signaling pathway, *EGFR* tyrosine kinase inhibitor resistance, and focal adhesion. *ITGA2* is involved in the phagosome, the PI3K–Akt signaling pathway, focal adhesion, and ECM–receptor interaction, which are all enriched in the comparison of Ningxiang pigs vs. Berkshires (Figure 6).

### 3.6. Validation by qRT-PCR

**Figure 7:**
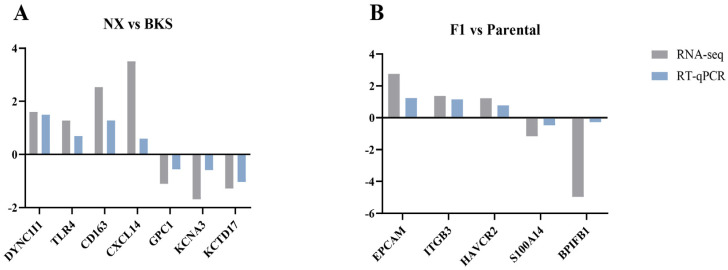
qRT-PCR validation of DEGs and their correlation with the RNA-seq data. X-axis, gene names; Y-axis, log_2_ (ratio relative expression).

To verify the accuracy of the RNA-seq data, we screened seven differential genes (*DYNC1I1*, *TLR4*, *CD163*, *CXCL14*, *GPC1*, *KCNA3*, and *KCTD17*) based on immune-related terms and pathways between Ningxiang pigs and Berkshires for qRT-PCR analysis. In F_1_ offspring and parental groups, we screened five differential genes (*EPCAM*, *ITGB3*, *HAVCR2*, *S100A14* and *BPIFB1*) for qRT-PCR analysis based on immune-related terms and pathways. Since the number of terms and pathways associated with each set we screened varied, the number of differential genes screened varied, resulting in a different number of genes in our validation. The findings revealed that the expression patterns of these genes by qRT-PCR were aligned with the RNA-seq data, confirming the reliability of the DEGs identified by RNA-seq in this study (Figure 7).

## 4. Discussion

Indigenous Chinese pig breeds have not been subjected to high intensity selection, thus maintaining genetic diversity in the existing population, and so their disease resistance is relatively strong [6,7,8]. Cytokines are important mediators of immune and inflammatory responses [9]. To investigate the differences of cytokines among pig breeds, we detected physiological and biochemical parameters in plasma, as well as certain immune indicators in the spleen of Ningxiang and Berkshire pigs and their F_1_ offspring. The levels of immune indicators, such as IgA, IL-6, and IFN-γ, in Ningxiang pig plasma are significantly higher than that in Berkshires. IgA is an important indicator for assessing the humoral immune response in animals [10]. It mediates humoral immunity and plays a role in preventing bacterial invasion and mucosal defense [11]. IL-6 and IFN-γ are crucial cytokines with pro-inflammatory properties. They can trigger an inflammatory response through various signaling pathways and enhance disease resistance [12,13]. In the spleen, the levels of IL-6 and IFN-γ are significantly higher in Ningxiang pigs compared with Berkshires.

IL-4 exerts anti-inflammatory effects on monocytes and macrophages [14,15,16,17]. Furthermore, research has shown that IL-4 enhances antigen-specific humoral responses [18,19]. Inflammatory conditions have been associated with an increased number of mast cells (MCs) in the spleen, which produce IL-4 in response to IgE and crosslinked antigens [20]. The level of IL-4 in the spleen of Ningxiang pigs is significantly higher than that in the other two pig breeds, indicating that the spleen of the Ningxiang pigs may contribute to the immune response. IL-17 is an important proinflammatory cytokine that activates NF-κB, which triggers the expression of proinflammatory cytokines and chemokines [21,22]. Our study found that the level of IL-17 in the spleen of Ningxiang pigs is significantly higher than that in the other two pig breeds, indicating that IL-17 might be crucial in innate immunity in the spleen.

The plasma IgA levels of Ningxiang pigs and F_1_ offspring are significantly higher than in Berkshire pigs. In another study, Tongcheng pigs were reported to have higher immunoglobulin content compared with foreign breeds [23], which is consistent with these findings, reflecting the difference in immune levels between Ningxiang and Berkshire pigs. As important pro-inflammatory cytokines, IL-6 and IFN-γ can trigger inflammatory responses through various signaling pathways and enhance disease resistance [12,13]. Compared with Berkshires, plasma IL-6 is significantly higher in Ningxiang pigs and F_1_ offspring. This may affect the immunity of Ningxiang pigs and F_1_ offspring by triggering more inflammatory reactions. Moreover, IFN-γ has an essential effect in both innate and adaptive immune responses to viral infections and intracellular bacterial challenges [24]. The levels of IFN-γ have been analyzed in different pig breeds, revealing that indigenous Chinese breeds have a better balance of T lymphocytes and immune capabilities [25]. The plasma IFN-γ level in Ningxiang pigs is significantly higher than in Berkshires and F_1_ offspring, which might indicate that the immune capacity of Ningxiang pigs is more stable than that of Berkshires and F_1_ offspring.

Plasma biochemical indexes can reflect the immune function of the host [26]. The levels of plasma ALB (albumin), and ALT (alanine aminotransferase), in Berkshires are significantly higher than those in Ningxiang pigs and F_1_ offspring. The activity of ALT increases in pigs during bacterial infection, which helps to enhance disease resistance [27]. The increase in ALB levels can enhance the immunity of piglets [28]. Additionally, ALB also plays a protective role for immunoglobulins. This finding is inconsistent with research [29], which may be due to differences in breeds and geographical locations. ALP (alkaline phosphatase) can promote the differentiation of T lymphocytes and the secretion of immunoglobulins, such as IgG and IgM, to affect the humoral immune response [10]. The levels of ALP in F_1_ offspring are significantly lower than that in the parents. Glucose (GLU) is associated with activation of the porcine spleen immune system and the increase in glucose intake [30]. The plasma glucose level of Ningxiang pigs is significantly higher than in Berkshires and F_1_ offspring. Our study shows that specific differences exist in the biochemical indicators of different breeds, which is consistent with previous research.

The splenic function encompass the elimination of senescent red blood cells, iron recycling, capturing and neutralizing pathogens, and initiating adaptive immune responses [4,31]. To explore the immunological mechanisms of different pig breeds and hybrid pigs, we conducted high-throughput transcriptome sequencing on pig spleens. Compared with Berkshires, 698 genes showed higher expression in Ningxiang pigs. Some of these highly expressed genes are enriched in immune-related pathways, such as scavenger receptor activity.

Scavenger receptors are cell surface receptors that interact with a variety of ligands. They function through mechanisms such as endocytosis, phagocytosis, adhesion, and signal transduction, ultimately degrading or removing harmful substances [32]. For instance, *CD163* is one of the markers for M2-type macrophages and is highly expressed in the lymph glands, spleen, and liver [33]. It functions as a critical receptor for the Porcine Reproductive and Respiratory Syndrome Virus (PRRSV) [34]. Moreover, *CD163* plays a crucial role in the innate immunity of pigs [35]. Notably, the anti-inflammatory cytokine IL-6 (interleukin-6) strongly upregulates *CD163* mRNA in monocytes and macrophages [36]. In this study, the IL-6 content in Ningxiang pigs was significantly higher than in Berkshires, which may be associated with the significantly higher *CD163* mRNA content in Ningxiang pigs. The macrophage receptor with collagenous structure (*MARCO*) belongs to the class A family of scavenger receptors and functions in immune regulation or lymphocyte development [37]. Specifically, *MARCO* is prominently expressed on the surface of macrophages in the marginal zone of the spleen, where it can bind to bacteria and promote phagocytosis and activation of immune responses [38]. The expression of *MARCO* is higher in Ningxiang pigs than in Berkshires. The variation in the expression of scavenger receptor family members may have a close relationship with phagocytosis, as well as the activation and transmission of immune signaling pathways.

The chemokines, as a major category of chemotactic molecules, recruit immune cells to the site of inflammation, participating in the inflammatory response and promoting immune reactions and wound healing [39]. Differential genes were identified in this study, such as *CXCL14* and *PPBP*, which are primarily enriched in the chemokine signaling pathway. This pathway participates in distinct functional roles, recruiting immune cells to initiate the inflammation and adaptive immune responses [40]. These highly expressed chemokines can recruit more immune cells to defend against pathogens, giving Ningxiang pigs a greater chemotactic immune cell capacity. *CXCL14* (chemokine (C-X-C motif) ligand 14) primarily contributes to immune cell migration and antibacterial immunity [41], which is crucial in the host defense against viral infections by enhancing the innate immune response [42]. *PPBP* (chemokine (C-X-C motif) ligand 7), also known as *CXCL7*, represents the most abundant α-granule protein [43] and plays an important role in inflammation. It targets basophils, eosinophils, natural killer (NK) cells, and neutrophils [44]. Therefore, *CXCL14* and *PPBP* likely play a significant role in chemokine receptor binding and affect immune regulation. We observed low expression of *CCL19* (chemokine (C-C motif) ligand 19), which performs a vital role in the migration, activation, expansion, and survival of T-cells involved in antiviral responses. Suppression of the *CCL19* axis results in T-cell dysfunction during viral infection [45].

In this study, we also identified hub genes with high (such as *ITGA2* and *TLR4*) and low expression (such as *EGFR*) through DEG and PPI network analysis. These hub genes participate in various immune pathways, such as phagosomes.

Phagosomes play a vital role in the processes of inflammation and protection against infectious agents [46]. Notably, the phagosome pathway is the most prominent in this group. The PI3K–Akt signaling pathway is closely associated with inflammation and can lead to the polarization of macrophages into M1 and M2 types (especially M1), as well as the production of pro-inflammatory cytokines such as TNF-α and IL-6 [47]. *ITGA2* (integrin α2) is typically involved in the recruitment of immune cells in immune responses, and these cells play a crucial role in regulating innate immunity [48]. Additionally, *ITGA2* contributes to the regulation of anti-inflammatory responses [49]. *TLR4* (toll-like receptor 4) operates through cooperation with interferon regulatory factors to stimulate the production of type I interferons (IFNs), enhancing defense against pathogens [50,51]. Additionally, the *TLR4* gene indirectly strengthens adaptive responses mediated by T cells and NK cells and promotes immune responses [52]. The expression of *ITGA2* and *TLR4* is higher in Ningxiang pigs compared with Berkshires. The high expression levels of hub genes may play a role in the immune response and could potentially enhance the defense against pathogens, thereby promoting the immune capacity. *EGFR* (epidermal growth factor receptor) is primarily associated with focal adhesion and the PI3K–Akt signaling pathway. *EGFR* is also involved in receptor signaling that activates the NF-κB pathway, leading to the expression of genes/transcription factors that contribute to B-cell ontogeny, autoimmunity, immune responses, and immunoglobulin production [53]. Inhibition or lack of the *EGFR* leads to a significant reduction in NF-κB pathway signaling [54]. The cytokines in this pathway are closely related to humoral immunity. The expression level of the *EGFR* is lower in Ningxiang pigs than in Berkshires. This low expression may potentially be related to inhibiting certain host immune signaling pathways and the ability to process antigens.

Compared with the parental pigs, *BPIFB1* and *DDX3X* show higher expression in F_1_ offspring, which are associated with immunity. The DEGs associated with immunity with lower expression in F_1_ offspring include *HAVCR2*, *CCR5*, and *ITGB3*, which are primarily concentrated in immune-related terms and pathways, such as the defense response, positive regulation of NF-κB signaling, pattern recognition receptor signaling pathways, and cell surface and cell adhesion molecule binding.

The innate immune system acts as the primary line of defense against pathogens [55]. For instance, *BPIFB1* (BPI fold-containing family B member 1) has a dual role in host defense and anti-inflammatory properties. Furthermore, *BPIFB1* shares structural similarities with Permeability Increasing Protein (BPI) proteins and LPS-binding proteins, indicating its contribution to innate immunity [56]. Among these three pig breeds, the expression level of the *BPIFB1* gene is highest in F_1_ offspring and lowest in Ningxiang pigs. *DDX3X* (DEAD box polypeptide 3) has a nucleotide sequence that fully complements the seed region of miR-181. Notably, miR-181 and its target genes are closely associated with immune regulation [57], suggesting that *DDX3X* is also associated with immune regulation. Among the three pig breeds, the *DDX3X* expression level is highest in F_1_ offspring and lowest in Berkshires. These highly expressed DEGs may be more effective in activating immune-related pathways and regulating the immune system. Adhesion molecules are essential signal transducers in the immune system. Signal transduction is facilitated by their involvement in transmitting signals from the external milieu of the cell into its intracellular domain [58,59]. *ITGB3* (integrin subunit β3) is enriched in this pathway and plays a crucial role in coordinating immune responses, which is widely distributed in the spleen. Previous research has emphasized the positive correlation between *ITGB3* and porcine PRRSV infection and transmission [60,61,62]. Among the three pig breeds, the expression level of the *ITGB3* gene is highest in Ningxiang pigs and lowest in F_1_ offspring. The expression of this gene may potentially impact the antigen presentation and processing capabilities. *CCR5* (chemokine receptor C-C chemokine receptor type 5) is present in various cell populations within the immune system, such as macrophages [63]. The modulation of inflammatory responses and immune-related functions is significantly influenced by *CCR5* [64,65]. It is noteworthy that pro-inflammatory factors, such as IL-2 [66] and TNF-α, also play a crucial role [67], and INF-γ [68] can increase the expression of *CCR5* in peripheral blood mononuclear cells. Among the three pig breeds, the *CCR5* expression level is highest in Berkshire pigs and lowest in F_1_ offspring. The difference in the expression levels of *CCR5* may affect inflammatory responses and immune regulation.

## 5. Conclusions

In this study, immune-related DEGs were identified through transcriptomic analysis of the spleen from native Chinese pig breeds, European native Berkshire pig, and their hybrids. According to GO enrichment, the genes *CD163, MARCO, CXCL14, CCL19, PPBP, BPIFB1, HAVCR2, DDX3X, CCR5*, and *ITGB3* are associated with immunity. KEGG results showed that the genes *EGFR, ITGA2*, and *TLR4* are significantly enriched in immune-related pathways. *EGFR* and *ITGA2* were identified as key genes in Ningxiang and Berkshire pigs through the analysis of DEGs and protein–protein interaction (PPI) networks. In conclusion, this study provides experimental evidence for the differences in immunological traits and molecular regulation between indigenous Chinese breeds and hybrid pigs.

## Figures and Tables

**Table 1:** Detection range and sensitivity of physiological indicators.

Index	Range	Sensitivity
IL-2	10~320 pg/mL	1.0 pg/mL
IL-4	2~64 pg/mL	0.1 pg/mL
IL-6	25~800 pg/mL	1.0 pg/mL
IL-17	1.5~48 pg/mL	0.1 pg/mL
IgA	25~800 µg/mL	1.0 µg/mL
TNF-α	6.25~200 pg/mL	1.0 pg/mL
IFN-γ	1.25~40 pg/mL	0.1 pg/mL

**Table 2:** Primers for quantitative real-time PCR.

Gene	Primer Sequence (5′-3′)	Annealing Temperature (°C)	Amplicon Length (bp)	GenBank No.
CD163	F: ATAGAGCTTGATGGGTCCGAGG	60	321	XM_021091123.1
	R: GCTCAACATTCTTGGGTCCTGG			
DYNC1I1	F: GCTGGGGAAAGGAAGTGCTA	60	186	XM_021102406.1
	R: GTCAGACATGCTGGCTTCCT			
TLR4	F: CAGATAAGCGAGGCCGTCATT	60	113	NM_001293316.1
	R: TTGCAGCCCACAAAAAGCA			
CXCL14	F: GAATTCATGAGGCTCCTGGCGGCCG	60	312	NM_001244128.1
	R: GGATCCCTATTCTTCGTAGACCCT			
GPC1	F: CTTAGTGCTGCTTTGCTTTTCAT	60	155	XM_047443961.1
	R: AGGGTTATTATGGGGTGGACTT			
KCNA3	F: GAGCTGCACTGACCAGTAGG	60	607	XM_021086763.1
	R: GTGCTGGCAGATGGATCACT			
KCTD17	F: GAGCTCACACAGATGGTATCC	60	95	XM_021091531.1
	R: TGGTCCTCACTCCCATAGTT			
ITGB3	F: CACGGGCAAGTACTGTGAGT	60	123	NM_214002.1
	R: TAGAAGCCAGTCCAGTCGGA			
S100A14	F: AAACTGGAGCTGTCTGTCGG	60	173	NM_001190168.1
	R: TGTAAATGCCCAAGCACCCA			
EPCAM	F: TGCTCTTTGAATGCGCTTGG	60	172	NM_214419.1
	R: AGAGCCCATCGTTGTTCTGG			
HAVCR2	F: TTCGACGGGAGCAGTAAAGC	60	105	XM_003134109.4
	R: CAAGGGCAGGACACAGTCAA			
BPIFB1	F: ACTCTTCGTGTCCAGTTTCTACT	60	159	NM_001101032.1
	R: TTCTGGACCGAGCCTGAGA			
β-Actin	F: GCGTAGCATTTGCTGCATGA	60	252	XM 003357928.4
	R: GCGTGTGTGTAACTAGGGGT			

**Table 3:** Statistics of plasma and spleen immunity-related physiology indicators in different pig breeds.

Items	(Mean ± SD)		
	Ningxiang	Berkshire	F_1_ Offspring of Ningxiang and Berkshires Pigs (F_1_ Offspring)
**plasma**			
IgA (ug/mL)	732.87 ± 55.55 ^a^	653.13 ± 24.41 ^b^	726.54 ± 68.17 ^a^
IL-2 (pg/mL)	308.57 ± 23.18 ^a^	307.30 ± 22.41 ^a^	277.48 ± 20.83 ^b^
IL-6 (pg/mL)	806.59 ± 42.16 ^a^	751.83 ± 54.18 ^b^	768.85 ± 35.15 ^b^
IFN-γ (pg/mL)	36.96 ± 2.94 ^a^	33.31 ± 4.44 ^b^	37.64 ± 3.76 ^a^
**spleen**			
IL-4 (pg/mL)	60.32 ± 4.45 ^a^	55.91 ± 4.56 ^b^	57.01 ± 5.02 ^b^
IL-6 (pg/mL)	755.00 ± 19.54 ^a^	740.01 ± 16.52 ^b^	750.82 ± 14.54 ^a,b^
IL-17 (pg/mL)	43.69 ± 2.41 ^a^	40.77 ± 3.27 ^b^	41.10 ± 4.75 ^b^
IFN-γ (pg/mL)	42.01 ± 1.86 ^a^	38.94 ± 3.21 ^b^	40.95 ± 1.53 ^a^
TNF-α (pg/mL)	195.08 ± 9.82 ^b^	203.01 ± 12.99 ^a^	207.59 ± 19.45 ^a^

In the same row, values with different small letter superscripts mean significant difference (*p* < 0.05), while with the same or no letter superscripts mean no significant difference (*p* > 0.05). The same as below.

**Table 4:** Statistics of plasma immunity-related biochemistry indicators in different pig breeds.

Items	(Mean ± SD)		
	Ningxiang	Berkshire	F_1_ Offspring
ALB (g/L)	60.02 ± 2.27 ^b^	70.86 ± 9.55 ^a^	60.66 ± 3.99 ^b^
ALT (U/L)	59.04 ± 11.48 ^b^	88.09 ± 23.96 ^a^	55.91 ± 13.58 ^b^
ALP (U/L)	31.29 ± 22.19 ^a^	27.57 ± 30.39 ^a^	5.29 ± 4.27 ^b^
GLU (mmol/L)	11.89 ± 2.27 ^a^	9.53 ± 1.86 ^b^	9.84 ± 3.15 ^b^
CHOL (mmol/L)	3.35 ± 0.59 ^a^	2.90 ± 0.63 ^b^	2.63 ± 0.27 ^b^
LDL-C3 (mmol/L)	2.24 ± 0.35 ^a^	1.78 ± 0.72 ^b^	1.56 ± 0.24 ^b^

## Data Availability

The datasets involved in this study can be found in online repositories. They would be accessed on 21 November 2024. The names of the accession number(s) can be found at: https://submit.ncbi.nlm.nih.gov/subs/bioproject/SRP473659.

## References

[1] Jiang Y.Z., Zhu L., Tang G.Q., Li M.Z., Jiang A.A., Cen W.M., Xing S.H., Chen J.N., Wen A.X., He T. (2012). Carcass and meat quality traits of four commercial pig crossbreeds in China. Genet. Mol. Res..

[2] Li B., Yang J., He J., Peng X., Zeng Q., Song Y., Xu K., Ma H. (2021). Characterization of the whole transcriptome of spleens from Chinese indigenous breed Ningxiang pig reveals diverse coding and non-coding RNAs for immunity regulation. Genomics.

[3] Mebius R.E., Kraal G. (2005). Structure and function of the spleen. Nat. Rev. Immunol..

[4] Bronte V., Pittet M.J. (2013). The Spleen in Local and Systemic Regulation of Immunity. Immunity.

[5] Morawietz G., Ruehl-Fehlert C., Kittel B., Bube A., Keane K., Halm S., Heuser A., Hellmann J. (2004). Revised guides for organ sampling and trimming in rats and mice—Part 3. A joint publication of the RITA and NACAD groups. Exp. Toxicol. Pathol..

[6] Clapperton M., Bishop S.C., Glass E.J. (2005). Innate immune traits differ between Meishan and Large White pigs. Vet. Immunol. Immunopathol..

[7] Liang W., Li Z., Wang P., Fan P., Zhang Y., Zhang Q., Wang Y., Xu X., Liu B. (2016). Differences of immune responses between Tongcheng (Chinese local breed) and Large White pigs after artificial infection with highly pathogenic porcine reproductive and respiratory syndrome virus. Virus Res..

[8] Wang J., Wang Y., Wang H., Wang H., Liu J.-F., Wu Y., Guo J. (2016). Transcriptomic Analysis Identifies Candidate Genes and Gene Sets Controlling the Response of Porcine Peripheral Blood Mononuclear Cells to Poly I:C Stimulation. G3 Genes Genomes Genet..

[9] Choi I.-S., Collisson E.W., Maheswaran S.K., Yoo H.S. (2002). Evaluation of cytokine gene expression in porcine spleen cells, peripheral blood mononuclear cells, and alveolar macrophages by competitive RT-PCR. FEMS Immunol. Med. Microbiol..

[10] Hou G., Peng W., Wei L., Li R., Huang X., Yin Y. (2021). Probiotics and *Achyranthes bidentata* Polysaccharides Improve Growth Performance via Promoting Intestinal Nutrient Utilization and Enhancing Immune Function of Weaned Pigs. Animals.

[11] Porter P. (1969). Transfer of immunoglobulins IgG, IgA and IgM to lacteal secretions in the parturient sow and their absorption by the neonatal piglet. Biochim. Biophys. Acta.

[12] Han Z., Liu Y., Wang G., He Y., Hu S., Li Y., Shi W., Wu J., Wang S., Liu H.T. (2015). Comparative Analysis of Immune Responses in Pigs to High and Low Pathogenic Porcine Reproductive and Respiratory Syndrome Viruses Isolated in China. Transbound. Emerg. Dis..

[13] Zhao Y., Cooper D.K.C., Wang H., Chen P., He C., Cai Z., Mou L., Luan S., Gao H.J.X. (2019). Potential pathological role of pro-inflammatory cytokines (IL-6, TNF-α, and IL-17) in xenotransplantation. Xenotransplantation.

[14] Standiford T.J., Strieter R.M., Chensue S.W., Westwick J., Kasahara K., Kunkel S.L. (1990). IL-4 inhibits the expression of IL-8 from stimulated human monocytes. J. Immunol..

[15] te Velde A.A., Huijbens R.J., Heije K., de Vries J.E., Figdor C.G. (1990). Interleukin-4 (IL-4) inhibits secretion of IL-1 beta, tumor necrosis factor alpha, and IL-6 by human monocytes. Blood.

[16] Gautam S., Tebo J.M., Hamilton T.A. (1992). IL-4 suppresses cytokine gene expression induced by IFN-gamma and/or IL-2 in murine peritoneal macrophages. J. Immunol..

[17] Hart P.H., Jones C.A., Finlay-Jones J.J. (1992). Interleukin-4 suppression of monocyte tumour necrosis factor-alpha production. Dependence on protein synthesis but not on cyclic AMP production. Immunology.

[18] Kim J.J., Yang J.S., Manson K.H., Weiner D.B. (2001). Modulation of antigen-specific cellular immune responses to DNA vaccination in rhesus macaques through the use of IL-2, IFN-gamma, or IL-4 gene adjuvants. Vaccine.

[19] Zhang X., Li G., Gao L., Mu L., Zhang L., Cong Y., Ding Z. (2013). Positive inductive effect of IL-18 on virus-specific immune responses induced by PRRSV-GP5 DNA vaccine in swine. Res. Vet. Sci..

[20] Toyoshima S., Wakamatsu E., Ishida Y., Obata Y., Kurashima Y., Kiyono H., Abe R. (2017). The spleen is the site where mast cells are induced in the development of food allergy. Int. Immunol..

[21] Miossec P., Kolls J.K. (2012). Targeting IL-17 and TH17 cells in chronic inflammation. Nat. Rev. Drug Discov..

[22] Song X., Qian Y. (2013). IL-17 family cytokines mediated signaling in the pathogenesis of inflammatory diseases. Cell Signal..

[23] Liu B., Zhang Q.D., Li K. (2005). Studies on Some lmmune Traits of Tongcheng, Swedish Landrace, BritishLarge White and Their Three-way Crossbred Pigs. Acta Vet. Zootech. Sin..

[24] Schoenborn J.R., Wilson C.B. (2007). Regulation of interferon-gamma during innate and adaptive immune responses. Adv. Immunol..

[25] Liu Y., Xu J., Fu W., Weng Z., Niu X., Liu J., Ding X., Zhang Q. (2012). Tissues Expression, Polymorphisms of IFN Regulatory Factor 6 (IRF6) Gene and Their Associated with Immune Traits in Three Pig Populations. Asian-Australas. J. Anim. Sci..

[26] Wang K., Zhou M., Gong X., Zhou Y., Chen J., Ma J., Zhang P. (2022). Starch-protein interaction effects on lipid metabolism and gut microbes in host. Front. Nutr..

[27] Oltean M., Gavrea R., Dumitrache M., Băguţ T., Gherman C.M., Cozma V., Györke A. (2012). Characterization of host-parasite interactions during the experimental *Trichinella spiralis* infection in pigs. Helminthologia.

[28] Zhao K., Yin H., Yan H., Tang W., Diao H., Wang Q., Qi R., Liu J. (2023). Dietary Supplementation of *Lactobacillus johnsonii* RS-7 Improved Antioxidant and Immune Function of Weaned Piglets. Animals.

[29] Ding Y.Y., Zhu M.Y., Ding J. (2020). Comparison and Analysis of Serum Biochemical indexes between Huoshou Black Pig and Berkshire Pig x Huoshou Black Pig at Different Days. Southwest China J. Agric. Sci..

[30] Deutz N.E.P., Reijven P.L.M., Athanasas G., Soeters P.B. (1992). Post-operative changes in hepatic, intestinal, splenic and muscle fluxes of amino acids and ammonia in pigs. Clin. Sci..

[31] Lewis S.M., Williams A., Eisenbarth S.C. (2019). Structure and function of the immune system in the spleen. Sci. Immunol..

[32] Xiang X., Zhang Y., Li Q., Wei J., Liu K., Shao D., Li B., Olszewski M.A., Ma Z., Qiu Y. (2020). Expression profile of porcine scavenger receptor A and its role in bacterial phagocytosis by macrophages. Dev. Comp. Immunol..

[33] Wang F., Qiu H., Zhang Q., Peng Z., Liu B. (2012). Association of two porcine reproductive and respiratory syndrome virus (PRRSV) receptor genes, CD163 and SN with immune traits. Mol. Biol. Rep..

[34] Burkard C., Opriessnig T., Mileham A.J., Stadejek T., Ait-Ali T., Lillico S.G., Whitelaw C.B.A., Archibald A.L. (2018). Pigs Lacking the Scavenger Receptor Cysteine-Rich Domain 5 of CD163 Are Resistant to Porcine Reproductive and Respiratory Syndrome Virus 1 Infection. J. Virol..

[35] Alvarez B., Martínez P., Yuste M., Poderoso T., Alonso F., Domínguez J., Ezquerra A., Revilla C. (2014). Phenotypic and functional heterogeneity of CD169^+^ and CD163^+^ macrophages from porcine lymph nodes and spleen. Dev. Comp. Immunol..

[36] Buechler C., Ritter M., Orsó E., Langmann T., Klucken J., Schmitz G. (2000). Regulation of scavenger receptor CD163 expression in human monocytes and macrophages by pro- and antiinflammatory stimuli. J. Leukoc. Biol..

[37] Aruffo A., Bowen M.A., Patel D.D., Haynes B.F., Starling G.C., Gebe J.A., Bajorath J. (1997). CD6—Ligand interactions: A paradigm for SRCR domain function?. Immunol. Today.

[38] Kraal G., van der Laan L.J., Elomaa O., Tryggvason K. (2000). The macrophage receptor MARCO. Microbes Infect..

[39] Charo I.F., Ransohoff R.M. (2006). The many roles of chemokines and chemokine receptors in inflammation. N. Engl. J. Med..

[40] Carvalho F.A., Nalbantoglu I., Aitken J.D., Uchiyama R., Su Y., Doho G.H., Vijay-Kumar M., Gewirtz A.T. (2012). Cytosolic flagellin receptor NLRC4 protects mice against mucosal and systemic challenges. Mucosal Immunol..

[41] Lu J., Chatterjee M., Schmid H., Beck S., Gawaz M. (2016). CXCL14 as an emerging immune and inflammatory modulator. J. Inflamm..

[42] Zlotnik A., Yoshie O. (2012). The Chemokine Superfamily Revisited. Immunity.

[43] Kanikarla Marie P., Fowlkes N.W., Afshar-Kharghan V., Martch S.L., Sorokin A.V., Shen J.P., Morris V.K., Dasari A., You N.Y., Sood A.K. (2021). The Provocative Roles of Platelets in Liver Disease and Cancer. Front. Oncol..

[44] Laing K.J., Secombes C.J. (2004). Chemokines. Dev. Comp. Immunol..

[45] Förster R., Davalos-Misslitz A.C., Rot A. (2008). CCR7 and its ligands: Balancing immunity and tolerance. Nat. Rev. Immunol..

[46] Blander J.M., Medzhitov R. (2004). Regulation of phagosome maturation by signals from toll-like receptors. Science.

[47] Cui W., Zhou S., Wang Y., Shi X., Liu H. (2021). Cadmium exposure activates the PI3K/AKT signaling pathway through miRNA-21, induces an increase in M1 polarization of macrophages, and leads to fibrosis of pig liver tissue. Ecotoxicol. Environ. Saf..

[48] Liang W., Ji L., Zhang Y., Zhen Y., Zhang Q., Xu X., Liu B. (2017). Transcriptome Differences in Porcine Alveolar Macrophages from Tongcheng and Large White Pigs in Response to Highly Pathogenic Porcine Reproductive and Respiratory Syndrome Virus (PRRSV) Infection. Int. J. Mol. Sci..

[49] Gao Y., Wahlberg P., Marthey S., Esquerré D., Jaffrézic F., Lecardonnel J., Hugot K., Rogel-Gaillard C. (2012). Analysis of porcine MHC using microarrays. Vet. Immunol. Immunopathol..

[50] Mair K.H., Sedlak C., Käser T., Pasternak A., Levast B., Gerner W., Saalmüller A., Summerfield A., Gerdts V., Wilson H.L. (2014). The porcine innate immune system: An update. Dev. Comp. Immunol..

[51] Su M., Hu R., Tang T., Tang W., Huang C. (2022). Review of the correlation between Chinese medicine and intestinal microbiota on the efficacy of diabetes mellitus. Front. Endocrinol..

[52] Park B.-J., Ahn H.-S., Han S.-H., Go H.-J., Kim D.-H., Choi C., Jung S., Myoung J., Lee J.-B., Park S.-Y. (2021). Analysis of the Immune Responses in the Ileum of Gnotobiotic Pigs Infected with the Recombinant GII.p12_GII.3 Human Norovirus by mRNA Sequencing. Viruses.

[53] Pathak S.K., Kumar A., Bhuwana G., Sah V., Upmanyu V., Tiwari A.K., Sahoo A.P., Sahoo A.R., Wani S.A., Panigrahi M. (2017). RNA Seq analysis for transcriptome profiling in response to classical swine fever vaccination in indigenous and crossbred pigs. Funct. Integr. Genom..

[54] Hardbower D.M., Singh K., Asim M., Verriere T.G., Olivares-Villagómez D., Barry D.P., Allaman M.M., Washington M.K., Peek R.M., Piazuelo M.B. (2016). EGFR regulates macrophage activation and function in bacterial infection. J. Clin. Investig..

[55] Akira S., Uematsu S., Takeuchi O. (2006). Pathogen Recognition and Innate Immunity. Cell.

[56] Li J., Xu P., Wang L., Feng M., Chen D., Yu X., Lu Y. (2020). Molecular biology of BPIFB1 and its advances in disease. Ann. Transl. Med..

[57] Ninsuwon J., Waiyamitra P., Roongsitthichai A., Surachetpong W. (2020). Expressions of miR-155 and miR-181 and predictions of their structures and targets in pigs (*Sus scrofa*). Vet. World.

[58] Kumar A., Ouyang M., Van den Dries K., McGhee E.J., Tanaka K., Anderson M.D., Groisman A., Goult B.T., Anderson K.I., Schwartz M.A. (2016). Correction: Talin tension sensor reveals novel features of focal adhesion force transmission and mechanosensitivity. J. Cell Biol..

[59] Korytina G.F., Akhmadishina L.Z., Kochetova O.V., Aznabaeva Y.G., Zagidullin S.Z., Victorova T.V. (2019). The Role of Serum Amyloid A1, Adhesion Molecules, Chemokines, and Chemokine Receptors Genes in Chronic Obstructive Pulmonary Disease. Russ. J. Genet..

[60] Tang Q.-h., Zhang Y.-m., Xu Y.-z., He L., Dai C., Sun P. (2010). Up-regulation of integrin beta3 expression in porcine vascular endothelial cells cultured in vitro by classical swine fever virus. Vet. Immunol. Immunopathol..

[61] Li W., Wang G., Liang W., Kang K., Guo K., Zhang Y. (2014). Integrin β3 Is Required in Infection and Proliferation of Classical Swine Fever Virus. PLoS ONE.

[62] Yang C., Lan R., Wang X., Zhao Q., Li X., Bi J., Wang J., Yang G., Lin Y., Liu J. (2020). Integrin β3, a RACK1 interacting protein, is critical for porcine reproductive and respiratory syndrome virus infection and NF-κB activation in Marc-145 cells. Virus Res..

[63] Rottman J.B., Ganley K.P., Williams K.C., Wu L., Mackay C.R., Ringler D.J. (1997). Cellular localization of the chemokine receptor CCR5. Correlation to cellular targets of HIV-1 infection. Am. J. Pathol..

[64] Zhen Y., Wang F., Liang W., Liu J., Gao G., Wang Y., Xu X., Su Q., Zhang Q., Liu B. (2018). Identification of Differentially Expressed Non-coding RNA in Porcine Alveolar Macrophages from Tongcheng and Large White Pigs Responded to PRRSV. Sci. Rep..

[65] Kim S., Lim B., Mattoo S.u.S., Oh E.Y., Jeong C.-G., Kim W.-I., Lee K.-T., Lee S.-M., Kim J.-M. (2021). Comprehensive Transcriptomic Comparison between Porcine CD8^−^ and CD8^+^ gamma Delta T Cells Revealed Distinct Immune Phenotype. Animals.

[66] Wu L., Paxton W.A., Kassam N., Ruffing N., Rottman J.B., Sullivan N.J., Choe H., Sodroski J.G., Newman W., Koup R.A. (1997). CCR5 Levels and Expression Pattern Correlate with Infectability by Macrophage-tropic HIV-1, In Vitro. J. Exp. Med..

[67] Hariharan D., Douglas S.D., Lee B., Lai J.-p., Campbell D.E., Ho W. (1999). Interferon-gamma upregulates CCR5 expression in cord and adult blood mononuclear phagocytes. Blood.

[68] Patterson B.K., Czerniewski M.A., Andersson J., Sullivan Y.B., Su F., Jiyamapa D., Burki Z.G., Landay A.L. (1999). Regulation of CCR5 and CXCR4 expression by type 1 and type 2 cytokines: CCR5 expression is downregulated by IL-10 in CD4-positive lymphocytes. Clin. Immunol..

